# Risk, Resilience and Survival: Migrant Sex Workers on Accra's Oxford Street, Ghana

**DOI:** 10.1002/puh2.70353

**Published:** 2026-08-24

**Authors:** Sylvester Kyei‐Gyamfi, Frederick Hans Ampiah, Marian Kpakpah

**Affiliations:** ^1^ Department of Children Ministry of Gender Children and Social Protection Accra Ghana; ^2^ UNDP Country Office Freetown Sierra Leone; ^3^ Ministry of Gender Children and Social Protection Accra Ghana

**Keywords:** Ghana, migrant sex workers, occupational risk, resilience, social–ecological model, urban vulnerability

## Abstract

Although female sex workers in Ghana experience multiple occupational risks, limited research has examined how migration status shapes these vulnerabilities and coping strategies. This study investigates the occupational risks and resilience of migrant female sex workers operating along Oxford Street in Osu, Accra, to generate evidence on the distinctive influence of migration on their lived experiences. Guided by the social–ecological model (SEM), a qualitative descriptive design was employed. Twenty migrant female sex workers were recruited using purposive sampling supported by peer referral recruitment and interviewed in English, French or Hausa using a semi‐structured interview guide. The SEM informed the development of the interview guide and the interpretation of the findings, whereas the data were analysed using an inductive thematic approach. Interviews were transcribed verbatim and analysed thematically. Although participants experienced challenges commonly reported among female sex workers, migration status intensified vulnerability through language barriers, deportation threats, xenophobic discrimination, weak access to justice and the absence of family support networks. Participants nevertheless demonstrated resilience through peer solidarity, strategic identity management, spiritual coping and informal safety practices. The study contributes new evidence by demonstrating that migration functions as a structural determinant shaping vulnerability and resilience across multiple ecological levels. The findings underscore the need for migrant‐sensitive, rights‐based and non‐punitive interventions that strengthen protection while recognising migrants’ adaptive survival strategies.

## Graphical abstract

Using the social–ecological model, this qualitative descriptive study explores how migration shapes occupational risks and resilience among migrant female sex workers operating along Oxford Street in Accra, Ghana. The findings show that migration functions as a structural determinant of vulnerability, with violence, economic coercion, stigma, language barriers, police exploitation, legal precarity and fear of deportation interacting across individual, interpersonal, community and structural levels. Despite these challenges, participants demonstrated resilience through peer solidarity, client screening, identity management, spiritual coping and informal safety practices. The study highlights the need for rights‐based, migrant‐sensitive interventions that strengthen legal protection, institutional accountability, access to health and social services, and multisector collaboration to improve the safety, dignity and well‐being of migrant female sex workers.

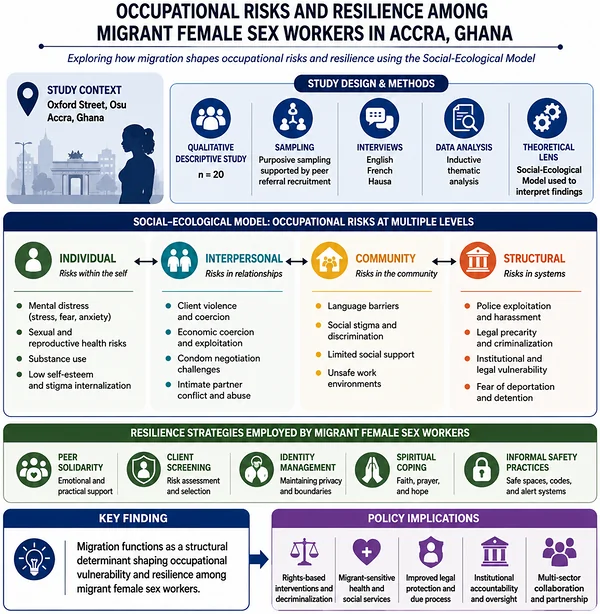

## 1 Introduction

Sex work is characterised by substantial occupational risks, including interpersonal violence, criminal victimisation, sexually transmitted infections (STIs), poor mental health, substance abuse, traumatic injuries and premature mortality [1]. Globally, female sex workers bear a disproportionate burden of HIV infection, violence and other adverse health outcomes due to structural inequalities, stigma, criminalisation and limited access to healthcare and social protection services [2, 3, 4]. In sub‐Saharan Africa (SSA), punitive legal environments, weak institutional protection and pervasive social stigma further exacerbate these vulnerabilities [2, 4]. For migrant female sex workers, these occupational risks are intensified by insecure immigration status, language barriers, disrupted family support systems, xenophobia and the constant threat of detention or deportation, all of which constrain access to justice and formal protection mechanisms.

Although sex work has been widely studied globally, research has predominantly focused on HIV prevention, condom use and sexual health, with comparatively limited attention given to how migration status shapes occupational vulnerability and resilience, particularly in low‐ and middle‐income countries. In Ghana, existing studies have substantially improved understanding of violence, HIV risk, condom negotiation, stigma, healthcare access and structural vulnerabilities among female sex workers [5, 6, 7]. However, these studies have generally treated female sex workers as a relatively homogeneous population, providing limited evidence on how migration status influences occupational risk, access to services, legal protection, social isolation and adaptive coping strategies.

Understanding migration is particularly important within the Ghanaian context because Accra has become an important destination for both internal and cross‐border migrants seeking livelihood opportunities in the informal economy [8, 9]. Oxford Street in Osu represents one of the city's busiest commercial and entertainment corridors, attracting local residents, tourists, expatriates and migrants from across West and Central Africa [10, 11]. Consequently, migrant female sex workers operating within this environment encounter occupational risks that arise not only from commercial sex work itself but also from the intersection of migration, legal insecurity and urban informality.

Ghanaian moral and cultural norms, reinforced by strong religious beliefs and persistent stigma surrounding commercial sex, continue to discourage open discussion of sexuality and contribute to the marginalisation of female sex workers [12, 13, 14, 15]. These norms often frame sex work as a moral rather than a public health or human rights issue, reinforcing discrimination and limiting public support for protective interventions. For migrant women, these challenges are compounded by foreign nationality, limited familiarity with local institutions, language barriers, disrupted family networks and insecure legal status, further restricting access to health, legal and social protection services.

Emerging evidence suggests that migration should not be viewed merely as a demographic characteristic but as a structural determinant that interacts with gender, occupation and legal status to shape exposure to violence, exploitation and social exclusion [2, 16]. Recent Ghanaian research among transnational sex workers similarly demonstrates that migrant women experience distinct forms of violence, intimidation and institutional exclusion associated with cross‐border mobility and irregular migration status [17]. Nevertheless, important gaps remain regarding how these migration‐related factors influence occupational risks, resilience and everyday survival strategies among migrant female sex workers operating within Ghana's urban commercial sex industry.

This study addresses these gaps by examining the lived experiences of migrant female sex workers operating along Oxford Street in Accra. Rather than treating migration as a background characteristic, the study conceptualises migration as a structural condition that interacts with individual, interpersonal, community and societal factors to shape occupational vulnerability and resilience. By applying the social–ecological model (SEM), the study extends existing Ghanaian scholarship beyond documenting occupational risks among female sex workers to explaining how migration reshapes these risks across multiple ecological levels.

Accordingly, the study sought to answer three research questions as follows:

1. What forms and levels of occupational risk do migrant female sex workers encounter along Oxford Street?
2. How does migration status shape these occupational risks?
3. What coping and resilience strategies do migrant female sex workers employ to navigate these risks?

Collectively, these questions seek to generate context‐specific evidence capable of informing migrant‐sensitive, rights‐based and non‐punitive policy responses that address both the structural determinants of harm and the adaptive strategies employed by migrant female sex workers in Ghana.

## 2 Theoretical Foundation: SEM

The present study is underpinned by SEM, which explains how health, behaviour and social outcomes arise through interactions between individuals and the multiple social, institutional and structural environments in which they live [17, 18, 19]. Originally developed by Bronfenbrenner and subsequently adapted within public health and violence research, SEM has become one of the most widely applied frameworks for understanding complex social problems because it recognises that vulnerability and resilience are shaped by interacting influences operating at multiple ecological levels rather than by individual behaviour alone [20, 21].

SEM was selected because migrant female sex workers experience multiple, interconnected forms of vulnerability that cannot be adequately explained by individual‐level factors alone. Their experiences are simultaneously influenced by migration status, legal environments, gender inequality, economic deprivation, workplace conditions, social relationships, community attitudes and institutional responses. Consequently, SEM provides an appropriate framework for examining how these interacting influences shape occupational risks, access to protection and resilience [2, 16, 22].

Within this study, SEM informed the development of the semi‐structured interview guide by ensuring that questions explored experiences across multiple ecological levels. During analysis, however, themes were generated inductively from participants’ narratives rather than being organised according to predetermined theoretical categories. Following the development and refinement of themes, SEM was subsequently applied as an interpretive framework to examine how the emergent findings interacted across the individual, interpersonal, community and structural levels. This approach preserved the inductive nature of the thematic analysis while ensuring theoretical coherence during interpretation [19, 23, 24].

The individual level focuses on participants’ personal characteristics and lived experiences, including migration histories, economic circumstances, language proficiency, health, substance use and personal coping strategies. The interpersonal level examines relationships with clients, peers, intimate partners, intermediaries and others who may either increase vulnerability or provide support and protection. The community level considers the influence of neighbourhood environments, workplace settings, social norms, policing practices, healthcare accessibility and local institutional arrangements on participants’ daily experiences. Finally, the structural level encompasses the wider legal, political, economic and cultural systems that shape migration, criminalisation, institutional protection, social exclusion and access to justice [2, 19, 25].

A major strength of SEM is its capacity to integrate these multiple ecological influences within a single analytical framework, thereby avoiding explanations that attribute vulnerability solely to personal behaviour or individual choice [26, 27]. This perspective is particularly valuable for understanding migrant female sex workers, whose experiences reflect the cumulative interaction of personal circumstances, social relationships, community environments and broader structural conditions.

Nevertheless, SEM has recognised limitations. Although it identifies multiple levels of influence, it does not specify the relative contribution of each level or prescribe explicit analytical mechanisms for examining interactions among them [28]. The framework has also been criticised for paying comparatively limited attention to historical processes, political economy and power relations that underpin structural inequalities. These limitations were considered during interpretation by grounding all findings in participants’ narratives and using SEM as an interpretive rather than deterministic framework.

Overall, SEM provides a coherent conceptual foundation for understanding how migration status interacts with gender, occupation, legal systems, community environments and social relationships to shape occupational risks and resilience among migrant female sex workers in Accra. By examining these experiences across multiple ecological levels, the framework supports a more comprehensive understanding of vulnerability and informs the development of multi‐level policy and practice interventions.

## 3 Methods

### 3.1 Study Design

This study employed a qualitative descriptive research design to explore the occupational risks, resilience strategies and lived experiences of migrant female sex workers operating along Oxford Street in Accra, Ghana. A qualitative descriptive approach was considered appropriate because it enabled the collection of rich, contextually grounded accounts of participants’ experiences while remaining close to their own descriptions of events and meanings [29, 30].

The study was informed by an interpretivist paradigm, which assumes that reality is socially constructed and that individuals assign meaning to their experiences within particular social and cultural contexts [31]. This perspective aligned with the study's objective of understanding how migrant female sex workers experienced and navigated occupational risks from their own viewpoints rather than testing predetermined hypotheses.

SEM served as the study's conceptual framework by informing the development of the semi‐structured interview guide and supporting interpretation of the findings across the individual, interpersonal, community and structural ecological levels [18, 19]. Thematic analysis remained primarily inductive, with SEM applied during interpretation rather than as a predetermined coding framework [23, 24].

Data collection and preliminary analysis occurred iteratively, allowing emerging issues to be explored in greater depth during subsequent interviews while maintaining the study objectives [24, 31]. The study is reported in accordance with the Consolidated Criteria for Reporting Qualitative Research (COREQ) to enhance transparency in describing the research team, participant recruitment, reflexivity, data collection and analytical procedures [32].

### 3.2 Study Setting

The study was conducted along Oxford Street in Osu, a major commercial and entertainment corridor within the Korle Klottey Municipal Assembly in the Greater Accra Region of Ghana. Osu is one of Accra's most cosmopolitan neighbourhoods, characterised by a concentration of restaurants, bars, hotels, shopping centres and nightlife that attracts local residents, tourists, expatriates and business travellers [11, 33, 34]. Its vibrant night‐time economy and high human mobility make Oxford Street one of Accra's busiest urban commercial corridors [33, 35].

Accra is Ghana's principal destination for internal and cross‐border migrants because of its diverse economic opportunities, particularly within the informal sector [8, 9, 33]. Consequently, Oxford Street has become a major hub for migrant female sex workers seeking livelihood opportunities in the city's night‐time economy. The study site was purposively selected because of its high concentration of migrant female sex workers from diverse national and cultural backgrounds, making it an information‐rich setting for exploring occupational risks and resilience [36]. The urban context also provided an opportunity to examine how environmental and structural conditions shape the occupational experiences of migrant female sex workers in Accra.

### 3.3 Research Team and Reflexivity

Qualitative research recognises that researchers are the primary instruments of data collection and interpretation; consequently, their backgrounds, experiences and professional positions may influence the research process [37, 38]. Recognising this, deliberate reflexive strategies were employed throughout the study.

The principal researcher is a male qualitative researcher and social scientist with extensive experience conducting research among vulnerable and hard‐to‐reach populations in Ghana, including studies involving migration, gender, HIV and child protection. At the time of the study, the principal researcher was professionally affiliated with MoGCSP. Two trained research assistants, who had previous experience in qualitative field research, supported the fieldwork after receiving additional training on the study objectives, qualitative interviewing techniques, research ethics, confidentiality, informed consent and interviewing vulnerable populations [32, 36]. Collectively, the research team was able to conduct interviews in English, French, and Hausa, enabling participants to communicate in their preferred language without relying on external interpreters [39, 40].

The researchers recognised that their professional backgrounds and institutional affiliations could influence participant trust and disclosure. Consequently, participants were informed that the study was being conducted in an independent research capacity rather than as a government investigation and that participation would not affect their immigration status, access to social services or relationships with any public institution. Initial access through trusted community gatekeepers and peer networks further facilitated rapport and helped minimise potential power imbalances during interviews [32, 37, 41]. Neither the principal researcher nor the research assistants had any prior relationship with the participants before recruitment.

Reflexivity was maintained through regular debriefing meetings following interviews, reflexive field notes documenting contextual observations and emerging analytical reflections, and continual comparison of developing interpretations with participants’ narratives. These procedures helped ensure that the findings remained grounded in participants’ lived experiences rather than researchers’ assumptions [24, 37, 38, 42].

### 3.4 Participant Recruitment and Sampling

Participants were recruited using purposive sampling supported by peer referral recruitment. Participants were recruited using purposive sampling supported by peer referral recruitment. Purposive sampling enabled the deliberate selection of migrant female sex workers with direct experience of the phenomenon under investigation, whereas peer referrals facilitated safe access to this hidden and highly stigmatised population through trusted community networks [36, 43, 44]. To maximise variation in experiences, participants were purposively selected from different national backgrounds, age groups, living arrangements, lengths of residence in Ghana and occupational experiences [31, 36].

Eligible participants were female migrants aged 18 years or older who were actively engaged in commercial sex work along Oxford Street or its immediate environs and were willing to provide informed consent. Participant ages were verified during recruitment to ensure that only adults meeting the inclusion criteria were enrolled. Individuals who were unable to communicate in the interview languages or who were unable to provide informed consent because of alcohol or drug intoxication at the time of recruitment were excluded.

Participant recruitment was initiated through trusted community gatekeepers, who introduced the research team to initial participants. These participants subsequently referred other eligible migrant female sex workers who satisfied the inclusion criteria. Although peer referrals facilitated access to additional participants, participant selection remained purposive throughout the study to ensure diversity of experiences rather than relying solely on chain‐referral recruitment [43]. Before participation, the research team explained the study objectives, eligibility criteria, confidentiality measures, voluntary nature of participation and participants’ rights before obtaining written informed consent. Recruitment continued until sufficient informational power and thematic depth had been achieved and no substantially new insights relevant to the study objectives were emerging [45, 46].

### 3.5 Data Collection Procedures

Data were collected using semi‐structured, in‐depth interviews, an approach well suited to exploring participants’ lived experiences while allowing flexibility to probe emerging issues [36, 47]. The interview guide was developed from the study objectives and informed by SEM to explore factors operating at the individual, interpersonal, community and structural levels [18, 19]. Before fieldwork commenced, the guide was reviewed by researchers experienced in qualitative research and migration studies to improve its clarity, relevance and sequencing.

Interviews were conducted by the principal researcher with the support of two trained field assistants at locations that were safe, private and convenient for participants [41]. A common interview guide was used throughout the study to promote consistency, while allowing flexibility to probe participants’ responses where necessary. Interviews were conducted by the principal researcher, with support from the trained research assistants, in English, French or Hausa according to participants’ preferences. Conducting interviews in participants’ preferred language enhanced rapport and facilitated richer descriptions of sensitive experiences [39, 40].

With participants’ consent, all interviews were audio‐recorded, and field notes were taken to capture contextual observations and non‐verbal cues that might assist interpretation [42]. Interviews lasted approximately 30–45 min and were conducted at times and locations that were convenient and safe for participants, thereby minimising disruption to their livelihood activities while allowing sufficient opportunity to explore their experiences in depth [41]. Data collection continued until sufficient informational power and thematic depth had been achieved [45, 46]. The relatively short duration reflected the occupational realities of the participants, many of whom depended on nightly client solicitation for their livelihoods. Consequently, interviews were scheduled at mutually convenient times and locations to minimise disruption to their income‐generating activities while allowing sufficient opportunity to obtain detailed accounts of their experiences. Data collection continued until sufficient informational depth had been achieved and no substantially new information relevant to the study objectives was emerging [45, 46]. During the interviews, participants were encouraged to clarify or elaborate on their responses, and interview summaries were periodically restated to confirm that their views had been accurately understood [48].

### 3.6 Data Analysis

Interview recordings were transcribed verbatim, and interviews conducted in French and Hausa were translated into English before analysis. Translations were reviewed against the original recordings to preserve participants’ intended meanings and minimise loss of contextual meaning during translation [40].

The data were analysed using reflexive thematic analysis following the six‐phase approach described by Braun and Clarke [23, 24]. The research team first familiarised themselves with the transcripts through repeated reading before generating initial inductive codes directly from participants’ narratives. Related codes were subsequently organised into candidate themes, which were iteratively reviewed, refined, defined and named to ensure that they accurately reflected recurring patterns across the dataset and addressed the study objectives.

Coding was undertaken by the principal researcher and one additional qualitative researcher experienced in thematic analysis. Both researchers independently coded an initial subset of transcripts to compare code development and enhance analytical consistency. Coding differences were discussed during analytical meetings and resolved through consensus, consistent with the principles of reflexive thematic analysis, rather than through statistical measures of inter‐coder reliability [24, 49]. Following agreement on the developing coding framework, the remaining transcripts were analysed using the same analytical approach, with regular discussions held to refine categories, review emerging themes and ensure that interpretations remained grounded in participants’ narratives.

The analysis was primarily inductive. Following the development of themes, the SEM was applied as an interpretive framework to examine how the identified themes interacted across the individual, interpersonal, community and structural ecological levels. Consequently, the SEM informed interpretation rather than the initial coding process, preserving the inductive nature of the thematic analysis while ensuring theoretical coherence [19, 24]. Representative quotations were selected to illustrate each theme and strengthen the transparency and credibility of the findings [50].

### 3.7 Trustworthiness of the Study

The trustworthiness of the study was established using the criteria of credibility, dependability, confirmability and transferability [50]. Credibility was enhanced through the use of semi‐structured interviews, probing questions, audio‐recording and periodic clarification of participants’ responses during interviews [48]. Dependability was promoted through the use of a common interview guide and consistent data collection procedures, whereas field notes documented contextual observations and methodological decisions throughout the study [49]. Confirmability was strengthened by grounding the findings in participants’ narratives through repeated comparison of the emerging themes with the original transcripts and the use of representative quotations in Section 4. Transferability was supported by providing detailed descriptions of the study setting, participants and research procedures to enable readers to assess the applicability of the findings to similar contexts [51].

### 3.8 Ethical Considerations

Ethical approval for the study was obtained from the University of Environment and Sustainable Development Ethics Review Board before data collection commenced. Permission to conduct the study was obtained from the relevant authorities and community gatekeepers.

To safeguard participants’ privacy, no personally identifiable information was collected, and pseudonyms were used during transcription and reporting. Interviews were conducted in private locations chosen by participants, and all interview recordings and transcripts were securely stored and accessed only by the research team.

## 4 Results

### 4.1 Demographic Characteristics of Participants

The study comprised 20 migrant female sex workers operating along Oxford Street in Accra (see Table 1). Participants ranged in age from 18 to 35 years, with the majority aged 24–29 years (*n* = 9), followed by 18–23 years (*n* = 7) and 30–35 years (*n* = 4). All participants, therefore, met the study's eligibility criterion of being 18 years of age or older. All participants were migrants from West and Central Africa, comprising Nigerians (*n* = 8), Liberians (*n* = 3), Ivorians (*n* = 3), Togolese (*n* = 2), Congolese (*n* = 2) and Sierra Leoneans (*n* = 2).

**TABLE 1:** Demographic characteristics of respondents (*n* = 20).

Characteristic	Frequency (*n*)	Per cent
**Age range**		
18–23	7	35
24–29	9	45
30–35	4	20
**Nationality**		
Nigerian	8	40
Congolese	2	10
Togolese	2	10
Ivorian	3	15
Sierra Leonean	2	10
Liberian	3	15
**Living arrangement**		
Shared apartment with ‘madam’	11	55
Renting small room with 1–2 peers	5	25
Hotel‐based (escort model)	3	15
Street‐based, no fixed sleeping place	1	5
**Total**	20	100

Living arrangements reflected varying levels of social and economic vulnerability. More than half of the participants (*n* = 11) lived in shared apartments managed by a ‘madam’, an arrangement that provided some degree of protection but often required the payment of substantial commissions. Five participants rented small rooms with one or two peers, three primarily operated from hotels, whereas one participant reported having no fixed residence and slept on the streets, representing the most precarious living situation within the sample.

### 4.2 Nature and Extent of Risk Exposure on Oxford Street

Participants described working along Oxford Street as being associated with multiple and interconnected occupational risks. Their accounts highlighted experiences of physical and sexual violence, threats to personal safety, sexual health risks, substance use, police exploitation, spiritual fears, legal insecurity and language‐related barriers. Although the nature and severity of these risks varied, participants consistently portrayed their working environment as one characterised by uncertainty, vulnerability and limited protection. The findings are presented under eight sub‐themes corresponding to the major forms of risk reported by participants.

#### 4.2.1 Physical and Sexual Violence

Participants frequently reported experiences of physical and sexual violence while engaging in commercial sex work along Oxford Street. These incidents included physical assault, forced sexual acts, robbery and intimidation, most commonly occurring in hotel rooms or other secluded locations where assistance was difficult to obtain. Participants described these experiences as a routine occupational risk that heightened their sense of insecurity while working.

Some men will beat you and even take back their money after the work. If you shout, they tell people you are a thief or a foreigner causing trouble. [Age 24].

Violence is our daily bread here. A man can become a beast once the door is locked because he knows we have no family here to defend us. [Age 29].

#### 4.2.2 Extreme Occupational Vulnerability

Participants cited the threat of death as the most serious occupational danger linked with commercial sex work on Oxford Street. A frequently cited example was the death of a fellow sex worker known as *‘*Spendlove*’*, whose experience served as a constant reminder of the dangers associated with meeting unfamiliar clients. Participants also reported witnessing cases in which sex workers were abandoned after violent encounters, reinforcing their fears about personal safety.

We all heard about Spendlove. She went with a stranger, became seriously ill afterwards, and did not survive. Since then, every new client makes you afraid because you never know what may happen. [Age 22].

I have seen girls dumped from moving cars after being used. Some do not survive, and because we are migrants, their deaths are often ignored. [Age 31].

#### 4.2.3 Sexual Health and Economic Coercion

According to the participants, financial difficulties play a significant role in determining sexual health decisions. Although condom use was widely recognised as a means of protecting against STIs, some participants reported accepting requests for unprotected sex in exchange for higher payment. Others indicated that clients sometimes removed condoms without their consent or became aggressive when condom use was insisted upon.

Sometimes the hunger is too much. When a man offers you triple the money for skin‐to‐skin sex, you pray and accept because the money can solve many problems back home. [Age 19].

We try to use condoms, but some clients become aggressive and tear them off. If you argue too much, they may hurt you or even report your illegal stay. [Age 26].

#### 4.2.4 Substance Abuse and Dependency

The participants stated that they regularly used alcohol and other psychoactive substances at work. They explained that these substances helped them cope with the emotional demands of commercial sex work, reduce fear and prepare themselves to engage with clients. Some participants also described substance use as a means of managing feelings of shame and psychological distress associated with their occupation.

Before I go out, I take at least three sachets of strong gin to numb my mind. If I am sober, I cannot stand the way these men look at me or the things they want to do. [Age 21].

You need the weed or the pills to be brave on the street at night. Without the drugs, the shame of what you are doing will overwhelm you. [Age 33].

#### 4.2.5 Institutional Betrayal and Police Exploitation

Participants reported frequent interactions with law enforcement officers who they perceived as exploitative rather than protective. They described experiences of extortion, intimidation and demands for sexual favours in exchange for avoiding arrest or deportation. Some participants indicated that these encounters discouraged them from seeking police assistance, even when they had experienced violence or other forms of abuse.

The police are not our friends, although not all officers behave this way. Many take our money or force us to sleep with them for free, and when you refuse, they threaten to deport you. [Age 27].

When the police catch you, they do not care if a client has beaten you. They only care about what they can take from your pocket. [Age 25].

#### 4.2.6 Spiritual Fears and Ritual Vulnerability

Participants reported concerns about spiritual harm and ritual exploitation as part of their occupational experiences. Many expressed fears that some clients might use personal items, such as hair or used tissues, for ritual purposes, commonly referred to as *sakawa*. These beliefs influenced how participants assessed clients and contributed to feelings of fear and vulnerability while working.

You have to be careful with ‘strange’ clients who ask for your hair or used tissues. We fear they will use these things for rituals to get money. [Age 23].

Some clients come with their own charms. It feels as though they are trying to take your spirit and your luck, not just pay for sex. [Age 30].

#### 4.2.7 Deportation Threats and Legal Precarity as Mechanisms of Control

Participants described insecure immigration status and the constant fear of deportation as significant occupational risks that increased their vulnerability while working along Oxford Street. Several explained that some clients exploited their undocumented or irregular migration status by threatening to report them to immigration authorities whenever disagreements arose over payment, condom use or the services provided. Others indicated that the fear of immigration enforcement discouraged them from reporting violence, theft or other forms of abuse because they believed doing so might expose them to detention or deportation. These experiences illustrate how legal precarity functioned as a mechanism through which both clients and some authority figures exercised control over migrant sex workers.

Sometimes when you ask for your full payment, the man says he will call immigration because you are not from Ghana. At that point, you become afraid and leave without your money. [Age 24].

Even when somebody beats you, you just keep quiet because if the police start asking questions about your documents, you may end up being deported instead of getting justice. [Age 31].

#### 4.2.8 Language Barriers and Breakdown of Negotiation

Participants reported that language differences created additional challenges when interacting with clients, negotiating services and seeking assistance during emergencies. Those with limited proficiency in English or dominant Ghanaian languages indicated that communication difficulties often resulted in misunderstandings, reduced their ability to negotiate prices or insist on condom use and limited their capacity to report incidents of violence or exploitation.

Sometimes I cannot explain myself because I do not speak Twi or English well. Clients take advantage of that and pay less or refuse to honour the agreement. [Age 20].

When something bad happens, I cannot easily ask people for help because they do not understand my language, and I also struggle to understand them. [Age 28].

### 4.3 Coping and Resilience Mechanisms

Despite the risks associated with working along Oxford Street, participants described adopting a range of strategies to protect themselves and cope with the demands of their work. These strategies included relying on peer support, using substances to manage emotional distress, drawing on spiritual beliefs, concealing personal identities, assessing potential clients before accepting them and seeking protection from trusted intermediaries. The findings are presented under six sub‐themes.

#### 4.3.1 Sisterhood Circles and Shadow Monitoring

Participants described relying on fellow migrant sex workers for mutual protection and support. Informal networks, often organised around shared nationality or friendship, enabled them to monitor one another's movements, share information about potentially dangerous clients and provide assistance when someone failed to return as expected.

We have a system where we check the car number and tell a sister before we leave. If you stay too long, we start calling your phone or even go to the hotel to look for you. [Age 24].

Being a stranger is hard, so we migrants have to be our own family. If a sister is in trouble, we all come together to shout and shame the man until he pays or leaves her. [Age 29].

#### 4.3.2 Chemical Coping and Substance‐Mediated Resilience

Participants reported using alcohol and other substances to help them cope with the emotional demands of commercial sex work. They explained that substance use helped reduce fear, suppress distress and prepare them to interact with clients.

I drink about four sachets of strong bitters before I can even talk to a man. The alcohol gives me the ‘lion heart’ to stand there and ignore the insults from people passing by. [Age 22].

The work is too heavy for a sober mind. We use drugs to numb the shame so we can focus on making the money. [Age 19].

#### 4.3.3 Spiritual Coping Practices and Protective Beliefs

Participants described relying on prayer, protective charms, and other spiritual practices to help them feel safe while working. Many believed these practices offered protection from physical harm and ritual exploitation and provided reassurance when interacting with unfamiliar clients.

I have a small charm that I keep in my purse, which a mama gave me back home. It is for protection, so that no man can use my spirit for rituals or hurt me physically. [Age 26].

You cannot work this street with ordinary eyes. We use prayers and charms to attract good clients and keep the ‘sakawa’ boys far away from us. [Age 34].

#### 4.3.4 Identity Management and Strategic Concealment

Participants reported carefully controlling the personal information they shared with clients and others. Many used aliases, concealed their nationality or withheld details about their families to protect themselves from exploitation and to avoid exposing their work to relatives in their home communities.

I never use my real name or tell them which country I am truly from. If you tell them the truth, they can use it to track you or report you. [Age 21].

Back home, they think I am working in a big shop in Accra. I keep my two lives separate so that the shame of the street never reaches my mother or my children. [Age 30].

#### 4.3.5 Client Profiling and Informal Risk Assessment (‘Street Wisdom’)

Participants explained that they routinely assessed potential clients before agreeing to accompany them. Decisions were based on clients’ behaviour, appearance, level of intoxication and intuition developed through experience. Several participants referred to the death of a fellow sex worker, *Spendlove*, as an event that reinforced the need for caution.

After what happened to Spendlove, I do not go with men who look too wild or are in groups of three. My life is worth more than the extra cash. [Age 23].

You learn to watch the eyes and the way they talk before you enter the car. If my spirit says ‘no,’ I do not go, no matter how much money he is showing me. [Age 27].

#### 4.3.6 Reliance on Pimps and Informal Protection Arrangements

Some participants reported relying on pimps or locally based protectors to provide security, resolve disputes with clients and facilitate access to work opportunities. Although these arrangements offered protection, participants noted that protectors commonly received a share of their earnings and could themselves become a source of pressure.

Having a protector is a must if you want to work the busy spots. He makes sure no man leaves without paying, but at the end of the night, he takes part of my money as security fees. [Age 22].

The pimps are tough; they can fight off any area boy who tries to harass us, and sometimes they even find clients for us. But if you have a slow night and cannot pay them, they can become more dangerous than the clients. [Age 26].

## 5 Discussion

This study provides one of the few qualitative investigations focusing specifically on migrant female sex workers in Ghana and demonstrates that migration status fundamentally shapes both occupational vulnerability and resilience. Although many of the occupational risks identified are consistent with previous studies among female sex workers, the findings show that migration introduces additional structural constraints, including insecure legal status, language barriers, disrupted family support networks and fear of deportation, that interact across multiple ecological levels to influence women's everyday experiences of safety, health and survival. Consequently, migration should be understood not merely as a demographic characteristic but as a structural determinant of occupational vulnerability within the commercial sex industry [2, 16].

The findings demonstrate that the risks experienced by migrant sex workers on Oxford Street are not isolated events but are embedded within interacting structural, community, interpersonal and individual contexts. Interpreted through SEM, the study shows how vulnerability and resilience coexist across multiple ecological levels, shaping participants’ everyday experiences [16, 18].

At the structural level, persistent reports of police harassment, extortion and threats of deportation illustrate how legal and institutional systems can contribute to vulnerability rather than protection. These findings are consistent with recent Ghanaian evidence, which shows that violence against female sex workers remains a significant public health and human rights concern, with interactions involving police officers, first‐time clients and regular clients all contributing significantly to physical and sexual violence [6, 52]. Another set of national bio‐behavioural surveillance data from Ghana shows that violence undermines condom use and access to health services, whereas some female sex workers report providing sexual favours to law enforcement officers in order to avoid arrest, highlighting the structural consequences of criminalisation and weak institutional protection [6, 53].

Similar findings have been reported among migrant sex workers in Nigeria, South Africa and Kenya, where criminalisation, irregular migration status and weak legal protection increase exposure to violence and exploitation [2, 54, 55, 56, 57]. In the present study, participants described how their undocumented status was frequently used by both law enforcement officers and clients to intimidate or exploit them, suggesting that migrant status compounds vulnerabilities already documented among Ghanaian female sex workers by introducing additional fears of detention, deportation and reduced willingness to seek institutional support. These findings are consistent with recent qualitative evidence among transnational sex workers in Ghana. Inusah et al. [17] similarly reported that migrant women experienced violence, intimidation, exploitation and institutional exclusion associated with irregular migration status, while also expressing reluctance to seek formal assistance because of fears of arrest, deportation and social stigma. The present study extends this evidence by demonstrating how deportation threats, language barriers and disrupted family support networks interact across multiple ecological levels to shape occupational risks and adaptive coping strategies.

These findings are also consistent with recent qualitative evidence among transnational sex workers in Ghana. Inusah et al. [17] similarly reported that migrant women experienced violence, intimidation, social isolation and persistent fears associated with insecure migration status while working within the commercial sex industry. Their participants described limited access to formal protection, distrust of law enforcement and reluctance to report abuse because of fears relating to immigration enforcement and social stigma. The present study extends these findings by demonstrating how deportation threats, language barriers and disrupted family support networks interact across ecological levels to shape both occupational risks and adaptive coping strategies. Rather than viewing migration simply as a demographic characteristic, the current study illustrates how migration status functions as a structural determinant that amplifies vulnerability while simultaneously shaping resilience among migrant sex workers in Ghana [17, 58].

At the community level, Oxford Street emerged as a localised risk environment characterised by a vibrant nightlife economy, limited formal regulation and reliance on informal systems of control. Consistent with Rhodes’ [59] risk environment framework, these conditions create opportunities for violence, exploitation and unsafe working practices [60]. Participants’ concerns about ritual exploitation, particularly through narratives surrounding *sakawa*, further illustrate how locally embedded cultural beliefs influence perceptions of safety and decision‐making. Rather than dismissing these beliefs as superstition, previous studies have shown that they represent socially meaningful interpretations of risk within many West African urban settings [61, 62]. These findings also highlight the necessity of interpreting risk in Ghana's sociocultural context, where spiritual beliefs continue to influence perceptions of security, trust and interpersonal interactions, particularly among socially excluded groups.

At the interpersonal level, participants described frequent experiences of physical violence, payment refusal, condom sabotage and coercive negotiations with clients. These findings align with previous research conducted in Ghana, which revealed that client violence, reluctance to use condoms and financial incentives for engaging in unprotected intercourse persist as prevalent issues among female sex workers [6]. The 2020 Ghana Integrated Bio‐behavioural Survey indicated that, although the majority of female sex workers attempted to negotiate the use of condoms, significant obstacles to safer sexual practices remained, including client refusals and offers of increased payment for sex without condoms [53]. These findings are consistent with international evidence showing that unequal power relationships often shape transactional sexual encounters [63]. Studies from Ghana and India similarly demonstrate that women who are economically disadvantaged, socially marginalised or migrants have reduced bargaining power during client negotiations [5, 63]. The willingness of some participants to engage in condomless (‘skin‐to‐skin’) sex for higher payment further illustrates how economic necessity can influence sexual decision‐making [64]. The findings also reinforce evidence from the 2020 Ghana Bio‐behavioural Survey, which demonstrated that migrant and economically vulnerable female sex workers frequently experience constrained negotiating power in commercial sexual encounters [53]. By focusing specifically on migrant women, the present study demonstrates that immigration‐related vulnerabilities further weaken negotiating capacity beyond the economic pressures already documented among female sex workers generally.

At the individual level, participants demonstrated both vulnerability and resilience. Alcohol and psychoactive substances were commonly used to cope with fear, emotional distress and the psychological demands of sex work, a pattern also reported among street‐based sex workers in other settings [65]. Consistent with findings from Ghana, the use of alcohol and drugs has been identified as significant correlates of both physical and sexual violence among female sex workers. This reinforces the complex relationship between coping behaviours and occupational risk [6]. At the same time, participants actively developed strategies to enhance their safety, including peer‐support networks, identity concealment, client profiling, informal savings arrangements and spiritual coping practices. Similar forms of collective resilience have been documented among migrant and street‐based sex workers across diverse settings [56, 66]. The findings enhance the existing Ghanaian literature by showing that migrant women augment their coping strategies with adaptations specific to migration. These adaptations include concealing their identities, avoiding immigration authorities, heavily relying on co‐national networks and continuously assessing deportation risks during interactions with clients and law enforcement. However, reliance on pimps or informal protectors also exposed some women to financial exploitation and additional forms of dependency [67].

The findings also complement broader Ghanaian migration research demonstrating that migrant status influences access to healthcare, social support and protection services. Sznajder et al. [58] found that migration experiences significantly shape sexual and reproductive health vulnerabilities among migrant populations in Ghana. Consistent with this evidence, participants in the present study described how unfamiliarity with local institutions, language barriers, fear of immigration enforcement and limited family support constrained their ability to seek assistance following violence or exploitation. By focusing specifically on migrant female sex workers, this study extends previous Ghanaian research by illustrating how these migration‐related disadvantages intersect with occupational risks within the commercial sex environment.

Overall, the findings demonstrate that occupational vulnerability among migrant female sex workers is produced through dynamic interactions across structural, community, interpersonal and individual ecological levels rather than through individual behaviour alone. Criminalisation, insecure migration status, language barriers, social exclusion and weak institutional protection collectively create environments in which community‐level risks, unequal interpersonal relationships and individual coping strategies become mutually reinforcing. Although many occupational risks identified in this study are consistent with previous Ghanaian research involving female sex workers, the present findings show that migration status fundamentally reshapes these risks through legal precarity, disrupted social networks, restricted access to services and persistent fears of detention and deportation. The study, therefore, contributes new evidence by demonstrating how migration functions as a structural determinant of vulnerability and resilience, reinforcing the need for migrant‐sensitive, rights‐based and non‐punitive interventions that strengthen institutional accountability while recognising the adaptive survival strategies employed by migrant female sex workers in Ghana.

## 6 Conclusion

This study provides new qualitative evidence on the lived experiences of migrant female sex workers operating along Oxford Street in Accra and demonstrates that occupational vulnerability and resilience are shaped by interactions across multiple ecological levels. Participants described violence, economic coercion, exploitative policing, deportation threats, language barriers and ritual anxieties as important occupational risks, while simultaneously adopting diverse strategies, including peer support, identity management, client screening and spiritual coping, to enhance their safety and sustain their livelihoods.

By applying SEM, the study demonstrates that migration status is not merely a demographic characteristic but a structural condition that fundamentally shapes occupational risks, access to protection and opportunities for resilience. Migration‐related legal insecurity, disrupted social networks and limited institutional protection amplify vulnerabilities already associated with commercial sex work while simultaneously influencing the adaptive strategies employed by migrant women.

The study, therefore, contributes to the limited body of Ghanaian evidence on migrant female sex workers and highlights the importance of developing rights‐based, migrant‐sensitive and non‐punitive policies that strengthen institutional accountability, improve equitable access to health and social protection services and recognise the lived realities of migrant women working within urban informal economies.

## 7 Implications for Policy and Practice

The findings have several implications for policy and practice.

First, interventions should move beyond individual behaviour‐change approaches to address the structural conditions that increase vulnerability among migrant sex workers. Strengthening accountability for police misconduct, improving access to legal protection and ensuring that migrants can report violence without fear of immigration‐related consequences could enhance safety and access to justice.

Second, partnerships involving hotel operators, transport providers, community organisations and public health agencies could promote safer working environments through discreet reporting mechanisms, emergency referral pathways and locally appropriate safety protocols. Existing peer‐support networks identified in this study also provide an important platform for community‐based violence prevention and health promotion.

Third, health interventions should integrate psychosocial support with harm reduction, confidential STI and HIV services, trauma‐informed care and economic empowerment initiatives. Addressing financial insecurity may reduce dependence on high‐risk clients and strengthen women's capacity to negotiate safer working conditions.

Fourth, migration‐sensitive services should include multilingual information, interpretation support and accessible reporting mechanisms for migrants regardless of their documentation status. Such measures may reduce barriers to seeking assistance and improve access to essential services.

Fifth, future programmes should adopt participatory approaches that involve migrant sex workers in programme design, implementation and evaluation. Their lived experiences can help ensure that interventions are contextually relevant, acceptable and sustainable. Overall, the findings support multi‐level interventions consistent with the SEM that address both immediate safety needs and the broader structural factors shaping vulnerability.

Finally, given the increasing mobility of populations within the Economic Community of West African States (ECOWAS) sub‐region, policy responses should extend beyond national public health interventions to include regional collaboration on migrant health, access to justice and protection from exploitation. Strengthening partnerships among health services, immigration authorities, law enforcement agencies, civil society organisations and migrant‐led community groups may enhance coordinated responses that protect migrant female sex workers while respecting their dignity and human rights.

## 8 Limitations and Strengths

This study has several strengths. First, the application of SEM provided a comprehensive framework for examining how structural, community, interpersonal and individual factors interact to shape the experiences of migrant sex workers. This approach facilitated a more holistic understanding of vulnerability and resilience than would have been possible through an individual‐level perspective alone. Second, the qualitative descriptive design generated rich accounts of participants’ lived experiences, providing insights that are often overlooked in quantitative research. Conducting interviews in multiple languages and in participant‐selected locations enhanced trust and encouraged open discussion of sensitive issues. The inclusion of migrant women from different national backgrounds also broadened the range of perspectives represented in the study.

Several limitations should be acknowledged. Participants were recruited using purposive sampling supported by peer referrals, an approach that was appropriate for accessing this hidden and highly stigmatised population but may have excluded migrant women who were socially isolated or disconnected from existing peer networks. The relatively small sample and focus on a single urban setting limit the transferability of findings to other locations or forms of sex work. In addition, reliance on self‐reported experiences may have introduced recall or social desirability bias, particularly when discussing sensitive issues such as violence, substance use, undocumented migration and interactions with law enforcement. Finally, although SEM provided a useful framework for interpreting findings across multiple ecological levels, it does not determine the relative influence of each ecological level or fully account for broader historical and political processes shaping migration and social exclusion.

Despite these limitations, the study makes an important contribution to the limited literature on migrant sex workers in Ghana. By documenting how structural exclusion, migration status, occupational risks and resilience intersect within an urban setting, the findings provide an evidence base for developing more context‐sensitive, rights‐based and migrant‐responsive policies and programmes.

## Author Contributions

The authors conceived and designed the study, conducted data collection and analysis and drafted and revised the manuscript. The authors approved the final version for submission.

## Funding

The authors have nothing to report.

## Ethics Statement

Ethical approval was obtained from the University of Environment and Sustainable Development Ethics Review Board (Approval No. UESD/UEC/ECC/24/0003). All procedures adhered to internationally recognised ethical principles, including the Declaration of Helsinki, and complied with national and institutional guidelines governing research involving human participants.

## Consent

Written informed consent was obtained from all participants after they had been informed about the study objectives, the voluntary nature of participation, their right to withdraw at any time without penalty and the measures taken to protect their confidentiality.

## Conflicts of Interest

The authors declare no conflicts of interest.

## Data Availability

The qualitative data supporting the findings of this study are available from the corresponding author upon reasonable request. Access is subject to ethical considerations related to participant confidentiality and safety.
